# How Low Should You Go: Choice of Minimum Dose Prescription in Cranial Radiosurgery

**DOI:** 10.7759/cureus.282

**Published:** 2015-06-29

**Authors:** David Roberge, Claudie Leclerc-Champagne, Robert Doucet, Jan Seuntjens

**Affiliations:** 1 Department of Oncology, Division of Radiation Oncology, McGill University Health Center; 2 Department of Radiology, Radiation Oncology and Nuclear Medicine, University of Montreal; 3 Department of Radiation Oncology, Centre hospitalier de l'université de Montréal (CHUM); 4 Division of Medical Physics, McGill University Health Center

**Keywords:** radiosurgery, prescription, target dose, isodose

## Abstract

Background: In radiosurgery, the convention has been to prescribe radiation dose to a “covering isodose volume”. This is presumed to be the minimum dose received by the entire tumor. Our purpose was to review our practice, assess different means of specifying a prescription isodose, and test how reliable they were in the face of various calculation methodologies.

Methods: Different minimum doses were calculated using three calculation methods for 20 brain targets and compared to the original physician intent. Monte Carlo (MC) calculations were run down to 2% (1 sigma) statistics. Voxel size depended on the imaging field of view and the calculation engine. For pencil beam convolution ("finite size pencil beam" - FSPB) calculations with or without heterogeneity correction (Methods 1-2), the calculation grid matched the CT scan resolution. For MC calculations (Method 3), the highest available in-plane resolution was 256 x 256 pixels. The median voxel volume was thus 0.58 mm^3^ (0.47 to 0.95 mm^3^) for FSPB and 2.3 mm^3^ (1.87 to 3.80) for MC.

Results: The absolute minimum target dose varied substantially between the three calculation methods — up to 25% difference between Methods 1 and 3. The differences were reduced when comparing near-minimum doses with absolute minimal volumes ΔV, *D*PTV-ΔV. The median difference in the isodose covering the PTV-0.03 cm^3^ was 0% for Methods 1 and 2 and 3.6% for Methods 1 and 3. The median difference in the isodose covering the PTV-0.01 cm^3^ was 0% for Methods 1 and 2 and 2.2% for Methods 1 and 3.

In our data, the smaller the volume in which the minimum dose is calculated, the more sensitive this calculation was to dose calculation parameters. The standard deviation of the difference between physician intent and the isodose covering the PTV-0.01 cm^3^ was 2.9% (range from -3.3% to 9.3%).

Conclusion: In radiosurgery, absolute minimum doses are sensitive to changes in dose calculation grids and dose calculation algorithms. Based on our experience, standardizing dose prescription to the isodose volume covering the PTV-0.01 cm^3^ or the PTV-0.03 cm^3^ would have little impact on clinical practice and would be relatively insensitive to dose calculation parameters.

## Introduction

When tumors are treated with radiosurgery, the convention has been to prescribe the radiation dose to the “covering isodose volume”. This could be presumed to be the minimum dose received by the entire tumor. The reality of radiosurgery practice is that the prescription isodose does not typically encompass every voxel of the gross tumor volume (GTV) and the calculation of a precise minimum target dose is unreliable. This is true within the relatively homogeneous cranium and exacerbated when extracranial targets are encompassed by low-density tissue [1-3]. It thus is sensible to prescribe radiosurgery to an isodose volume that encompasses nearly all of the tumor. In some cases, this selection of the coverage isodose will be done by simple visual inspection. In others cases, the coverage isodose will be selected through the use of a dose-volume histogram [4]. The problem with such an approach is that it is subjective and does not allow consistent dose reporting. Here below are typical examples of a retrospective report and instructions in a prospective trial; in either case, the dose delivered could vary significantly depending on which “coverage isodose” is selected:

“The tumor volume was included within the 50% or greater isodose curve. The standard dose to the tumor margin was 25 Gy, but the doses were modified according to the tumor volume and shape” [5].

“The dose should be prescribed to the highest isodose line encompassing the clinical target volume [...] which can range from 50% to 90% of the maximum dose” [6].

Our purpose was to review our clinical practice, assess different quantitative means of specifying a prescription isodose, and test how reliable different types of prescriptions were in the face of varied calculation methodologies.

## Materials and methods

A CyberKnife radiosurgery system with fixed conical collimators has been used for cranial radiosurgery at our institution since 2009. Dose statistics for all radiosurgery patients are collected in a local database. These statistics for 2009 to 2013 were reviewed with special attention to radiosurgery prescription. During this period, cases were planned clinically using a pencil beam convolution ("finite size pencil beam" — FSPB) algorithm without heterogeneity correction.

Twenty consecutive cranial tumors (functional cases were excluded as the dose for these patients was prescribed to a point) treated in 2013 were selected for this work. This represented 13 patients treated for varied indications. The clinical prescriptions were defined by three different radiation oncologists.

For the 20 consecutive targets, the minimum target dose was retrospectively calculated with the three available dose calculation engines in the then current version of the Multiplan planning system (Multiplan 3.5.2, Accuray, Sunnyvale, California):

1. FSPB calculation without heterogeneity correction

2. FSPB with path length heterogeneity correction

3. Monte Carlo (2% statistics)

The voxel size for these calculations depended on the computed tomography scan field of view and on the calculation engine used. For the FSPB calculation, the calculation was performed at the native resolution of the CT scan. Monte Carlo calculations were limited to a 256 x 256 in-plane grid (with the native slice separation (1 mm) in the z-axis). The median voxel volume was thus 0.058 mm^3^ (0.047 to 0.095 mm^3^) for FSPB calculations and 0.23 mm^3^ (0.187 to 0.38 mm^3^) for Monte Carlo. A single voxel represented a median of 0.1% of the PTV volume for FSPB calculations (range: 0.005%-1%) and 0.5% (range: 0.02%-5%) of the PTV volume in the case of Monte Carlo.

For each target and calculation engine, the minimum PTV dose, the dose to 95% of the PTV (*D*_95_), and the PTV minus 0.1 cm^3^, 0.03 cm^3^, and 0.01 cm^3^ were evaluated using each of the three calculation methods.

## Results

In the period of June 24, 2009 to June 18, 2013, 708 patients were treated for 1,083 cranial targets. The median volume of these lesions was 1.14 cm^3 ^(range: 0.015 to 57.6 cm^3^). A median of 20 Gy (3.6 to 82 Gy) was prescribed to the 75% isodose (50% to 100%) covering 99.3% (34% to 100%) of the target.

In the 20 consecutive targets used for this work, the median volume was 0.7 cm^3^ (0.15 – 9.9 cm^3^). The most common diagnosis was haematogenous metastasis (16 lesions in nine patients) with the other four cases divided between two schwannomas, a meningioma, and a pituitary adenoma. As treated, the median prescribed dose was 20 Gy (range: 12 - 25 Gy). The median prescription isodose was 70% (range: 55 - 75%). The entire PTV volume was covered with a median of 96.2% of the prescribed dose (range: 88.9 - 100.9%).

When the plans were recalculated using pencil beam convolution with heterogeneity correction or a Monte Carlo algorithm, the reported minimum dose to the PTV changed in all cases. In every case, the minimum dose to a single voxel decreased when either form of heterogeneity correction was used. This decrease ranged from 0.3% of the prescribed dose to 26% of the prescribed dose. The median decrease using path length correction was 1.6% (range: 0.3-7%). The median decrease using Monte Carlo was 4.9% (range: 0.8-26%). These differences were substantially reduced when the near minimum dose concept was used instead of minimum dose (Table 1).

**Table 1:** Median change in minimum PTV dose as compared to standard FSPB calculation

	Path Length Correction	Monte Carlo
Minimum dose (last voxel)	-1.6% (-7% to -0.3)	-4.9% (-26% to -0.8%)
Near-minimum PTV-0.01 cm^3^	0% (-3.2% to 0%)	-3.6% (-11% to 0%)
Near-minimum PTV-0.03 cm^3^	0% (-2.9% to 0)	-2.2% (-9.5% to 0%)
Near-minimum PTV-0.1 cm^3^	0% (-3.2% to 2.9)	-1.7% (-9.5% to -2.9%)

**Figure 1:**
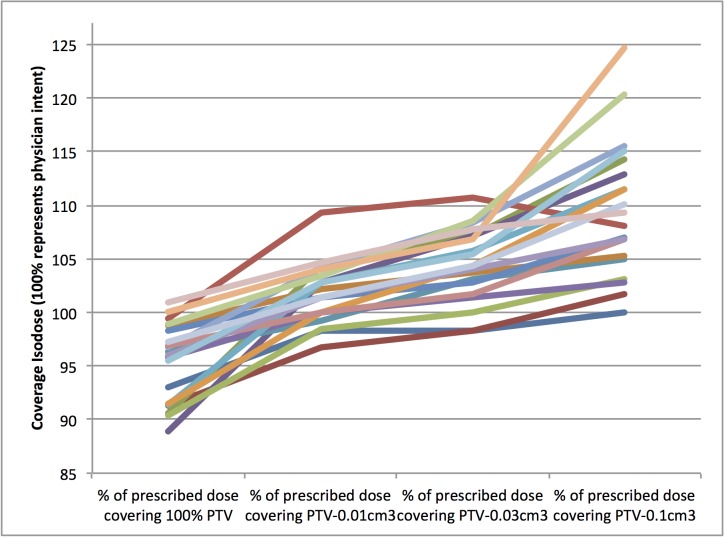
Physician intent vs. PTV coverage Which isodose covers the tumor using different potential minimum or near-minimum metrics is plotted relative to the real life dosimetry, as clinically approved by the physician (for example, the graph illustrates that clinical plans all excluded the last GTV voxel from the prescription isodose).

Using the same calculation parameters implemented clinically, the isodose volumes selected by the prescribing physicians were compared to four versions of near minimum dose: *V *- 0.1 cm^3^, *V* -0.03 cm^3^, *V *- 0.01 cm^3^, and *D*_95_. The standard deviation of the difference between physician intent and minimum dose parameter was smallest for *V *- 0.001 cm^3^ (2.9%, range from -3.3 to 9.3). Prescribing to cover *V *- 0.1 cm^3^, *V *- 0.03 cm^3^, *V *- 0.01 cm^3^, or *D*_95_ would have changed the delivered dose by a median of 6.3%, 3.4%, 2.9%, and 2.9% (Figure 1).

## Discussion

The lack of standardization in radiosurgery prescription arises in part from a lack of evidence linking dose prescription to clinical outcomes. The lack of detailed or consistent dose reporting may be partially to blame.

We have shown that the absolute minimum dose is not a reliable metric when one considers the dose calculation engine and dose matrix size. Unfortunately, limitations in our planning software did not permit us to completely disentangle the two variables. We have also shown in our clinic that using a near minimum of PTV as *V*_PTV_ – 10 mm^3^ would have little impact on our prescriptions. As cranial radiosurgery targets can be quite small (in our database of 1,083 cranial targets, 21% were smaller than 200 mm^3^), we showed that a near minimum dose based on an absolute, rather than relative, volume is more appropriate.

In the publication, “Standardization of terminology in stereotactic radiosurgery: Report from the Standardization Committee of the International Leksell Gamma Knife Society”, it is recommended that the minimum dose should be related to a specific tissue volume (for example, *D*_2%_ or preferably *D*_1 mm3_). The AAPM TG-101 report specifies the minimum and maximum dose to a “point”, with a “point” defined as 0.035 cm^3^ [1, 7].

We would argue that the specific minimum volume should not be left to the individual radiosurgery practitioner. For future scientific communication and evolution of patient care, it would be simpler if practitioners only varied the prescription dose and not the isodose coverage (volume). This would simplify the reporting of the dose prescribed to a group of patients. One has only to imagine reading the following two excerpts from a scientific abstract of the year 2020:

A. “Tumors were treated in a single fraction to doses ranging from 15 to 25 Gy prescribed to the 50-65% isodose volumes covering 80-100% of the target volume.”

or

B. “Tumors were treated in a single fraction to doses ranging from 8 to 27 Gy (dose to the target minus 10 mm^3^) prescribed to the 50-65% isodose volumes.”

The second abstract excerpt would be more informative to the reader. A table including each patient treated would need to be included in the manuscript associated with abstract A to convey the same information.

As the radiosurgery community may not be able to agree on a specific near minimum dose metric, it is still possible for each clinical practice to select its own. It is a caveat of this work that it may not reflect clinical practice in other clinical environments or with other radiosurgery devices.

## Conclusions

In radiosurgery, absolute minimum doses are sensitive to changes in dose calculation grids and dose calculation algorithms. Based on our experience, standardizing dose prescription to the isodose volume covering the PTV - 10 mm^3^ would have little impact on clinical practice and would be relatively insensitive to dose calculation parameters.
